# A low-variance subspace underlies individual differences in resting state fMRI

**DOI:** 10.64898/2026.01.25.701594

**Published:** 2026-01-27

**Authors:** Anastasia Borovykh, Max Weissenbacher, Stephanie Noble, Maxwell Shinn

**Affiliations:** 1 Department of Mathematics, Imperial College London, London, UK; 2 Machine Learning Lab, Capital Fund Management, Paris, France; 3 Department of Psychology, Northeastern University, Boston, US; 4 Department of Bioengineering, Northeastern University, Boston, US; 5 Institute for Cognitive & Brain Health, Northeastern University, Boston, US; 6 Department of Radiology & Biomedical Imaging, Yale Medical School, New Haven, US; 7 UCL Queen Square Institute of Neurology, University College London, London, UK

## Abstract

People differ remarkably from one another, yet isolating individual differences in their brain activity remains challenging. Non-invasive whole-brain recordings of human brain activity, such as those from resting state fMRI (rs-fMRI), are complex and noisy, making it difficult to isolate stable dimensions of individual differences. Ideally, we want to find a few core dimensions that vary across people but have high test-retest reliability, giving the same value each time they are measured in the same person. However, it is still unknown whether any such reliable dimensions exist, and if they do, what could drive this reliability. Here, we show that there is a low-dimensional linear subspace of highly-reliable rs-fMRI activity. These dimensions form personal fingerprints, allowing participants to be identified with high accuracy despite fingerprints explaining only a fraction of the total variance. Many of these dimensions inherit their reliability from a single morphological, demographic, or behavioral property, and most dimensions can be predicted from the anatomical layout of cortical regions. These dimensions were identified using reliability component analysis (RCA), a new dimensionality reduction technique similar to principal component analysis (PCA) but which maximizes reliability instead of explained variance. Together, our findings suggest that stable individual signatures can be isolated from rs-fMRI. These signatures reflect persistent anatomical and physiological differences, and provide a principled low-dimensional basis for biomarker discovery.

## Introduction

Resting state fMRI (rs-fMRI) is sensitive not only to population-level effects, but also to individual differences across participants (Elliott et al., 2019; Gratton et al., 2018; Noble et al., 2017). One critical property of trait-level individual differences is that they must have high test-retest reliability, giving the same result when measured in the same person on different occasions (Noble et al., 2021). Reliable individual differences in rs-fMRI activity likely reflect a combination of factors, including person-specific thought patterns (Geerligs et al., 2015), arousal fluctuations (Chang et al., 2016; Tagliazucchi and Laufs, 2014), autonomic processes (Soroka et al., 2025), intrinsic correlational structure (Noble et al., 2019), and variability in resting-state network organization and location (Bijsterbosch et al., 2019; Braga and Buckner, 2017; Kong et al., 2021). Systematic differences may also arise from anatomy, such as white matter pathways (Griffa et al., 2022; Honey et al., 2009) and brain volume (Pervaiz et al., 2020; Qing and Gong, 2016), as well as slowly-evolving traits such as age (Dosenbach et al., 2010) and disease states (Woo et al., 2017). Identifying reliable differences is essential for clinical applications (Lee et al., 2012).

However, we still do not understand the factors that underlie reliability in fMRI, and the extent to which they are driven by specific spatial regions, anatomical properties, demographic variables, or other characteristics. This is because reliability studies largely focus on quantifying and reporting the reliability instead of isolating it and tracing it back to its source. As a result, these existing studies rely on black-box methods such as hand-crafted statistics (Shinn et al., 2023) and complex matching algorithms (Finn et al., 2015). Ideally, we want to find a parsimonious number of independent dimensions of rs-fMRI activity that differ between people but are consistent across multiple scans of the same person.

Here, we identify a low-dimensional subspace of rs-fMRI activity that maximizes test-retest reliability. We find that linear combinations of simple timeseries statistics—such as the mean, standard deviation, and temporal autocorrelation—can be highly reliable, with independent dimensions having intraclass correlation coefficient (ICC) up to 0.98. This subspace serves as a low-dimensional fingerprint for each participant, allowing individual participants to be identified from the population with high accuracy, despite explaining only a fraction of the total variance in the original space. This subspace derives much of its reliability from brain morphology and physiology, and the dimensions naturally disentangle to represent individual sources. We release our method, reliability component analysis (RCA), as a scikit-learn–compatible library. Overall, our findings demonstrate that highly reliable dimensions can be isolated from an otherwise complex rs-fMRI signal, and that these dimensions bridge the relationship between structure and function.

## Results

### Reliability of functional brain metrics

**Figure 1:**
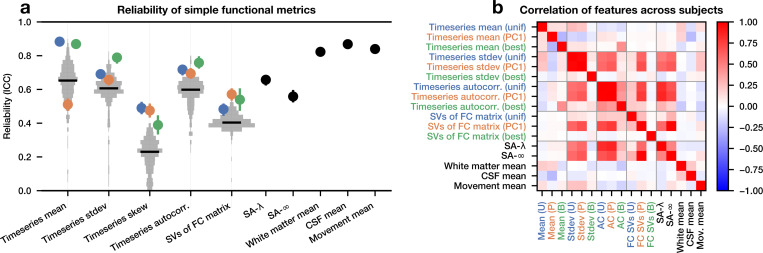
There are many reliable dimensions of rs-fMRI activity. (a) Metrics are evaluated for reliability using intraclass correlation coefficient (ICC). For metrics which produce a single value across the brain, the ICC and associated 95% confidence intervals are shown (all significant, p < floating point precision). For metrics which produce one value per region, a distribution of ICC for each region is shown, with a black line indicating the median. Overlaid is the ICC of the uniform projection of the regions (blue), the first principal component of the regions (orange), and the cross-validated best region (green). (b) The Spearman correlation of each of these features with each other.

First, we show that several simple statistics of rs-fMRI timeseries are reliable. We obtained four 15-minute scans from 999 participants from the Human Connectome Project (Sherif et al., 2014; Van Essen et al., 2013) acquired across two separate days. For each scan, we computed several simple timeseries features for each of 360 brain regions: the temporal mean, standard deviation, skew, and lag-1 temporal autocorrelation (Shinn et al., 2023). We also constructed features of the functional connectivity (FC) matrix using the first 360 singular vectors (84.8% explained variance on the training set, 77.4% on the test set). To quantify reliability, we used intraclass correlation coefficient (ICC), a measure of similarity within vs between groups which takes values from −1 to 1 (Koo and Li, 2016). We compute the ICC across participants, yielding one ICC per feature per region (or per singular vector for FC). We find that, consistent with previous work (Shinn et al., 2023), all of these features have highly significant ICCs (Figure 1a, grey). We compare these to other metrics previously shown to be reliable, including the measures of spatial autocorrelation SA-λ and SA−∞ (Shinn et al., 2023), as well as the “nuisance” parameters of head movement and mean white matter and cerebrospinal fluid (CSF) signal. We find that these other metrics and nuisance parameters also have high reliability. This indicates that many basic statistics derived from fMRI timeseries are preserved across different scans from the same individual.

If so many metrics and nuisance parameters have high reliability, does their reliability derive from the same underlying source? If so, we might expect that these metrics will be correlated across participants. We form three naïve proxies of each of the multivariate features: the average across regions (“uniform projection”), the first principal component (“PC1”), and the highest cross-validated ICC from each feature (“best region”). We compute the Spearman correlation across participants between each pair of these univariate metrics (Figure 1b) and find that for some features, the uniform projection and PC1 are correlated with each other, but for other features, they are not. Additionally, there is only a weak correlation between the “best region” projection and every other metric, despite all of them being very highly reliable on their own. This indicates that a mix of shared and independent sources of reliability can be found both within and across these features. Therefore, a specially-crafted metric combining these diverse sources of information might achieve even higher reliability than any of the naïve proxies.

### Maximizing reliability

**Figure 2:**
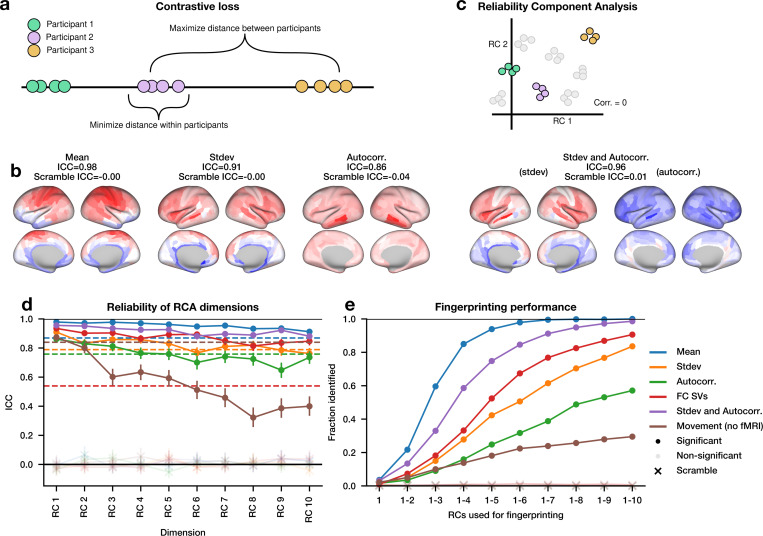
Reliability can be decomposed into linear components. (a) Schematic diagram of the contrastive loss function. (b) The correlation map for the most reliable dimension is plotted on the brain for each feature set of regional timeseries statistics. Intraclass correlation coefficient (ICC) for this dimension is computed using only participants in the test set. “Stdev and Autocorr.” concatenates these two feature sets and thus shows them plotted separately. (c) Schematic diagram of reliability component analysis (RCA). RCA minimizes the contrastive loss function for each dimension (reliable component, RC), sequentially under the constraint that no dimension correlates with existing dimensions. (d) The ICC for each dimension when RCA is performed on different feature sets. Points indicate ICC on the test set. For comparison, dotted lines indicate the ICC of the best cross-validated dimension from Figure 1a. “FC SVs” indicates a feature set constructed from the singular vectors of the functional connectivity matrix, and “Movement” indicates a feature set of within-scanner estimated movement parameters. Opacity indicates significance of ICC (p<.01 with Bonferroni correction). “x” indicates models trained with scrambled subject labels. (e) Fingerprinting performance using nearest-neighbor matching in multiple dimensions. The number of RCA dimensions used in the fingerprinting is shown on the x axis. Points indicate fingerprinting performance from the test set. Opacity indicates significance (p<.01 permutation test with Bonferroni correction)

To find a metric which maximizes reliability, we want to combine information from different brain regions into a single value for each scan. Each participant should have similar values for all of her scans, but different values from the scans of the other participants (Figure 2a). More precisely, this means finding a latent dimension in the feature space which minimizes within-participant distance but maintains a large variance across participants. To achieve this, we use a contrastive loss function, originally introduced in self-supervised machine learning (Chopra et al., 2005; Schroff et al., 2015) which found applications across neuroscience (Schmors et al., 2025; Schneider et al., 2023; Zhuang et al., 2021). Our method can perform both linear and nonlinear dimensionality reduction (see Methods), though here we focus on the linear case. We fit several of these models, one for each set of our best features: mean, standard deviation, autocorrelation, and FC SVs.

Our models identified a linear dimension for each feature set with high reliability. These dimensions had substantially higher cross-validated reliability than the best univariate features and had interpretable spatial patterns on the brain surface (Figure 2b). This is in contrast to identical models fit to scrambled subject labels, which had an ICC near zero (Figure 2b). We also fit a model on a rich representation of movement, containing all estimated rotations and translations with their first temporal difference, but found only a very small increase in ICC over the total magnitude of movement alone (Movement ICC=0.840, movement dimension ICC=0.874). While we tested both linear and non-linear models, the linear models were simpler and had slightly higher reliability on the test set (Figure S1).

We next asked if combinations of different feature sets could further improve reliability. We fit a model to each pair of feature sets. We found that the model combining standard deviation and autocorrelation improved ICC substantially compared to either feature alone (Figure 2b), whereas other models produced minimal to no improvement in ICC for the most reliable feature in the pair (Figure S2). Therefore, using a more diverse set of features may, but will not necessarily, increase the amount of reliable information.

### A maximally reliable linear subspace

We showed there is a highly reliable dimension in each set of features, but are there multiple such dimensions in the same set of features? To answer this, we developed an iterative procedure for identifying uncorrelated latent dimensions that are maximally reliable, which we call reliability component analysis (RCA). In RCA, the first reliable component (RC1) is the maximally reliable latent dimension, described in the previous section. Each subsequent reliable component (RC) is the most reliable latent dimension that is uncorrelated with all other RCs. While we only enforced decorrelation within the training set, RCs tend to be uncorrelated in the test set as well (Figure S3). Taking these dimensions together, RCA forms a maximally reliable subspace, where each scan occupies a single point in the subspace, and scans from the same participant form clusters (Figure 2c).

Many reliable dimensions exist across all tested sets of rs-fMRI timeseries features (Figure 2d). These dimensions, or RCs, often correspond to meaningful spatial structure, such as interior-exterior and anterior-posterior gradients, as well as loci around the temporal lobe (Figure 2b, Figure S4).

The dimensions of each subspace form a unique functional fingerprint of the participant. We performed a fingerprinting procedure inspired by (Finn et al., 2015). For each scan, we found its nearest neighbor, and then checked whether both scans were from the same participant. If so, we considered it a match. We computed the nearest neighbors in a space consisting of the first *N* RCs, with *N* ranging from 1 to 10. We found that for the Mean feature, we have near perfect fingerprinting on the test set using only 7 dimensions, and approximately 90% accuracy using only the first 10 dimensions for the FC model and the standard deviation + autocorrelation model (Figure 2e). By contrast, the ICC and fingerprinting performance for the first 10 principal components are low (Figure S5). Comparable fingerprinting performance has been obtained in previous work using 129,600 (Finn et al., 2015) or 360 (Larabi et al., 2021; Shinn et al., 2023) dimensions. Note that our fingerprinting is more difficult than in Finn et al. (2015) due to a larger sample size and the lack of a target-database split; applying the correlation-based method from Finn et al. (2015) to our data gives a fingerprinting accuracy of 24.6%.

The subspaces produced by RCA are robust to differences in methodology. A non-linear variant of RCA exhibits slightly worse reliability on the test set (Figure S4, Figure S6, Figure S1), but the non-linear RCA subspaces still show substantial overlap with their corresponding linear RCA subspaces (Figure S7). We also obtained similar subspaces when fitting using different random seeds, a non-iterative variant of RCA (see Methods), and a different contrastive loss function (Figure S7).

### Tracing the source of reliability

**Figure 3:**
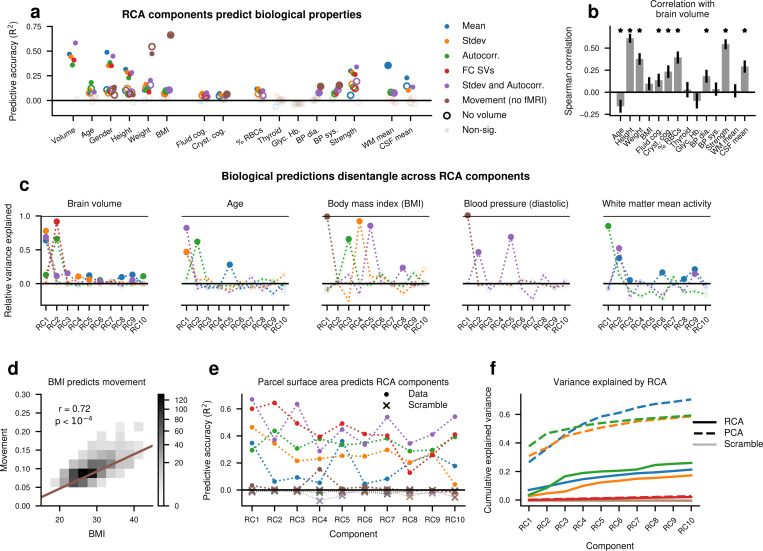
Reliable components reflect stable anatomical and physiological variables. (a) Reliable components predict anatomical, physiological, and behavioral variables on the test set (points). Y axis indicates the coefficient of determination $\left(R^{2}\right)$ for all variables except gender, which uses the pseudo- $R^{2}$ measure of explained deviance. When brain volume is regressed out (hoops), the predictive accuracy is no longer significant for some variables. Saturated color indicates permutation test p<.01 after Bonferroni correction. (b) Spearman correlation of brain volume with each of the structural or cognitive measures from (a), where * indicates significance (p<.001) with Bonferroni correction. (c) Predictive ability of individual reliable components, measured by $R^{2}$ of each given RC divided by the predictive power of the first 10 RCs together as shown in (a). Saturated color indicates permutation test *p* < .01 with Bonferroni correction. Only RCs that significantly predict the variable with at least 5% variance explained are shown. (d) Correlation between body mass index (BMI) and mean movement magnitude. (e) Predictive accuracy $\left(R^{2}\right)$ of participant-level surface area for each parcel in predicting each RC from multiple feature sets on the test set. “x” indicates models trained with scrambled subject labels. (f) Cumulative variance explained by RCs, compared to cumulative variance explained by principal components (PCs) and by RCs trained on scram-bled data, which all explain slightly less than 0 variance. All RCA components explain significantly more variance than chance (*p* < .01, one-sided permutation test).

Since our reliable subspaces are stable across repeated scans of the same participant, we asked whether they inherit this stability from other stable sources of individual variability, including anatomy, physiology, and demographics. First, we show that RCs predict these sources of variability using linear regression. We found that total brain volume, age, gender, height and weight, cognition, strength, and nuisance variables were consistently predicted with high accuracy by nearly all of the brain-derived RCs (*p* < .001) (Figure 3a, circles). Due to the high predictive accuracy of brain volume, we also tested whether the predictive accuracy of these other variables might be due to their relationship with brain volume (Figure 3b, *p* < .001) (Nazarzadeh et al., 2025; Pervaiz et al., 2020). When we adjusted for the effect of brain volume, we found still significant but attenuated predictive power for each (Figure 3a, hoops).

We next asked whether this predictive power was evenly distributed across components or sparsely concentrated in a few components. We tested each individual RC’s ability to predict total brain volume, age, body mass index (BMI), blood pressure, and white matter mean signal. We found that for each model, a small number of RCs provided high predictive accuracy, while the rest of the RCs provided little to no predictive power (Figure 3c). Brain volume and age were largely predicted by the same components, and blood pressure and BMI were partially predicted by the same components, but other properties were predicted by different components. For example, the first RC for autocorrelation predicts white matter activity, the second predicts brain volume and age, and the third predicts BMI, with other autocorrelation RCs offering little to no predictive power over these variables. The high predictive power of the movement feature on BMI was largely driven by the strong relationship between BMI and total movement magnitude (Figure 3d). We reiterate that RCA had no *a priori* knowledge of brain structure, physiological variables, or confounds, yet disentangled representations of these quantities emerged naturally by optimizing for reliable individual variation. We also note that some of these quantities, such as grip strength, did not disentangle across RCs (Figure S8). These results collectively suggest that the effects of many anatomical and physiological variables on rs-fMRI activity are confined to specific spatial patterns.

Finally, we asked whether the reverse is true: can measures of a participant’s local brain structure predict RCs? We used participant-level measurements of parcel surface area to predict each RC, and found significant prediction for nearly all RCs (*p* < .001), especially FC and Standard deviation + Autocorrelation (Figure 3e). By contrast, the RCs formed from movement parameters and from scrambled features could not be predicted by structure (Figure 3e). Therefore, parcel size is sufficient to predict many of the most reliable dimensions of rs-fMRI activity.

One possible trivial explanation of these results is that most of the variance is reliable, and thus, RCA captures most of the variance in the signal. To test this, we compared the cumulative variance explained by RCA to that explained by PCA, an upper bound on explainable variance. We found that RCA explains only a modest amount of variance, and often the first PC explains more variance than the first 10 RCs combined (Figure 3f). This confirms that RCA specifically captures patterns of brain activity which reliably encode individual differences.

## Discussion

We showed that individual variability in rs-fMRI can be captured in a low-dimensional, low-variance linear subspace. Patterns of rs-fMRI activity can be highly reliable, and the most reliable patterns are closely related to brain anatomy, physiology, and demographics. These characteristics often mapped onto specific components, allowing us to identify an underlying source from which each pattern of individual variability was inherited. In contrast to previous work (Dosenbach et al., 2010), the individual variability related to age was primarily coincident with brain volume, perhaps due to the narrow range of ages among our participants. Independent of brain volume, it was most strongly represented by autocorrelation features, perhaps due to the close connection between autocorrelation and aging (Grady and Garrett, 2013; Shinn et al., 2023).

We performed no preprocessing beyond the HCP minimal preprocessing pipeline. Notably, we did not adjust for nuisance regressors despite their high reliability (Pervaiz et al., 2020; Soroka et al., 2025), because we wanted to examine the relationship of these regressors with RCs (Xifra-Porxas et al., 2021). For instance, the mean white matter signal showed a strong overlap with the reliable subspace derived from the mean on the same components as total brain volume. By contrast, the subspace derived from head movement had minimal overlap with brain-derived reliable subspaces, indicating that reliability in head movement is not reflected in the reliability of rs-fMRI activity.

Our study revealed relationships between sources of individual variability and nuisance variables. For example, the signals within the white matter and CSF were highly reliable and minimally related to brain volume, but were largely manifest in the mean, with little impact on FC. Likewise, individual variability in head movement was highly reliable but unidimensional, and this reliability was almost fully explained by BMI, a relationship known to be heritable (Hodgson et al., 2017) and causal (Beyer et al., 2020).

An alternative interpretation of RCA is as an ideal linear subspace for clustering. Indeed, our fingerprinting performance indicates a strong clustering of participants. Unlike many linear dimensionality reduction methods, RCA does not focus on explaining variance, and indeed, explains only a modest fraction of the total variance in the data; in other words, high-variance components are not necessarily the most reliable (Rodriguez et al., 2025). Nevertheless, RCA is robust to different hyperparameters and contrastive loss functions, making it especially appropriate for complex datasets like rs-fMRI, where the signal of interest is a relatively small fraction of the total variance.

Not all features had many reliable dimensions. Notably, only a few RCs based on autocorrelation and movement had higher reliability than the best single feature, suggesting a simpler relationship between these features and reliability. In the case of autocorrelation, we found strong external correlates for each of the first three RCs. However, this was not the case for all sets of features. Future work with more sophisticated measures of brain structure and physiology can use RCA to further decompose reliability. For instance, nonlinear-RCA has the capacity to capture more nuanced patterns than linear-RCA, and larger datasets or data augmentation may help nonlinear-RCA capture more subtle patterns than linear-RCA. We anticipate that RCA will provide a smaller search space for functional biomarkers of slowly-progressing diseases.

## Methods

### Data

We used data from the Human Connectome Project (HCP) (Van Essen et al., 2013). HCP data are available through the HCP repository (https://www.humanconnectome.org/study/hcp-young-adult). Users must agree to data use terms before accessing ConnectomeDB; details are provided at https://www.humanconnectome.org/study/hcp-young-adult/data-use-terms. HCP investigators received IRB approval at the corresponding data collection sites. The University College London and Northeastern University Human Research Protection Programs approved secondary analysis of this dataset. We accessed the data using the CBRAIN project from McGill University (Sherif et al., 2014).

HCP data were preprocessed according to the HCP minimal preprocessing pipeline (Glasser et al., 2013). Data were parcellated into 360 regions using the Glasser parcellation (Glasser et al., 2016). All features were computed on the parcellated timeseries. Nuisance variables were not regressed out, and global signal regression was not performed. After removing incomplete scans, the dataset contained scans of 999 participants.

Participants received four resting state scans on two different days. We did not treat the two days separately in our analyses or in choosing matching pairs for the contrastive loss, as reliable differences between the two days within participants were minimal or absent. We included only participants who completed all four resting state scans, giving 999 participants. For the analyses correlating with structural and demographic variables, we excluded a small number of additional participants who refused or were unable to provide the given structural or demographic variable.

We split our data of 999 participants into a training set (749 participants) and a test set (250 participants) selected randomly with a fixed seed. All figures, analyses, and statistics shown are on the test set only, unless otherwise specified. The same training-test set split was used for all analyses.

Timeseries features were computed directly on the parcellated timeseries. The FC matrix singular vectors were computed by flattening the upper triangle (excluding the diagonal) of the FC matrix, and then performing SVD on the flattened matrices of the training set, using the resulting singular vectors to compute the scores for both training and test set participants. We computed the “best region” (or “best SV”) ICC for each feature by computing the ICC of each region or SV on the training set, finding the one with the maximum such value, and then reporting the ICC computed only on that region or SV within the test set.

Movement features included the estimated translation and angle (and their derivatives) in three axes, as well as the absolute magnitude, standard deviation, and temporal autocorrelation of each. It also included the mean, standard deviation, and autocorrelation of the total estimated movement magnitude timeseries, for a total of 51 values. The mean of the total estimated absolute displacement timeseries was the mean total movement magnitude.

### Reliability measure

To measure the reliability across scans, we used the intraclass correlation coefficient (ICC), a measure of the variance explained by participant identity compared to the total variance. We used the strictest variant of intraclass correlation coefficient, also known as “ICC(1,1)”, as our primary measure of reliability. We compute ICC following McGraw and Wong (1996) as

(1) $$\text{ICC}=\frac{\text{Va}{\text{r}}_{i}\left(E_{j}\left[Y_{i,j}\right]\right)R-E_{i}\left[\text{Va}{\text{r}}_{j}\left(Y_{i,j}\right)\right]}{\text{Va}{\text{r}}_{i}\left(E_{j}\left[Y_{i,j}\right]\right)R+E_{i}\left[\text{Va}{\text{r}}_{j}\left(Y_{i,j}\right)\right](R-1)},$$

where R is the number of repeated observations (here *R* = 4) and Y is an M×R matrix, with M the number of participants (here M=749 for the training set or M=250 for the test set).

### Contrastive loss

We trained our models using a contrastive loss function (Chopra et al., 2005). The contrastive loss function is designed to ensure that scans of the same participant (“positive” sample pairs) are located close to one another in the embedding space, while scans of different participants (“negative” sample pairs) are located far from one another in the embedding space. In other words, it optimizes for clustering positive pairs. Here, we use a one-dimensional embedding space. Let the set $P^{+}$ be the set of positive (i,j) pairs and the set $P^{-}$ be the set of negative (i,j) pairs, such that $(i,j)\in P^{+}$ indicates scans i and j are from the same participant, and $(i,j)\in P^{-}$ indicates they are from different participants. If we denote the vector of metrics from all brain regions of a particular scan by $x_{i}$ and the model under consideration by f , then our contrastive loss is given by,

$$L(f)=\frac{1}{\left|P^{+}\right|}\sum_{(i,j)\in P^{+}}{\left|f\left(x_{i}\right)-f\left(x_{j}\right)\right|}^{2}+\frac{1}{\left|P^{-}\right|}\sum_{(i,j)\in P^{-}}\max{\left(0,\epsilon -\left|f\left(x_{i}\right)-f\left(x_{j}\right)\right|\right)}^{2},$$

where the first expectation is taken over all positive pairs of vectors which belong to the *same* participant and the second expectation is conversely taken over all negative pairs which belong to *different* participants. The hyperparameter ϵ is a margin which is enforced between the same and different embeddings. Choosing ϵ larger will result in larger distances between the negative pairs. We note that the absolute value of points in the embedding space does not have an intrinsic interpretation; instead, only the relative distances between points capture the structure of the dataset. For our results shown here, we used ϵ=1.0 .

If the training set contains M participants with K≪M scans each, we have in total $MK(K-1)/2\approx MK^{2}/2$ positive pairs and $M(M-1)K^{2}/2\approx M^{2}K^{2}/2$ negative pairs. Therefore, there are approximately M=749 more negative sample pairs for each positive sample pair, creating a large asymmetry; we thus take the expectation of the positive and negative losses separately and with an optional weighting parameter to control for the differences in magnitude.

In practice, the loss is computed over a certain batch of scans which is randomly sampled from the training set. This batch size has been found to be a key limiting factor for performance of contrastive learning methods (Chen et al., 2022; Grill et al., 2020); using variations such as the triplet loss (Schroff et al., 2015) or using larger batch sizes tends to lead to better performance. The small size of the dataset used in the present study permitted the use of the entire training set in each batch, which in our experiments was essential to ensuring model training converged.

The model f is learned using gradient descent with the AdamW optimizer (Loshchilov and Hutter, 2017). We use a generous number of 2000 epochs with a learning rate of 10^−3^ to reach convergence on linear models, and 4000 epochs with learning rate 2 × 10^−4^ on non-linear models.

To verify robustness, we also ran simulations using an InfoNCE-style loss function, an alternative loss function frequently used in contrastive learning, given by

$$L(f)=\frac{1}{\left|P^{+}\right|\tau }\sum_{(i,j)\in P^{+}}\left|f\left(x_{i}\right)-f\left(x_{j}\right)\right|+\frac{1}{N}\sum_{i=1}^{N}\log\sum_{j=1}^{N}\exp\left(-\frac{\left|f\left(x_{i}\right)-f\left(x_{j}\right)\right|}{\tau }\right).$$

Typically, the similarity score is computed as the cosine similarity or dot product, scaled by a temperature τ . In our case the output f can be one-dimensional, so we use the norm instead. This loss is motivated by a cross-entropy objective: for a base embedding f(x) , pick the $f\left(x^{\prime }\right)$ such that it belongs to the same class, i.e., make the second element in the positive scan pair stand out compared to negative pair elements.

### Reliability Component Analysis (RCA)

Reliability Component Analysis is a novel technique to extract a maximally reliable linear or nonlinear subspace from a set of features, such as timeseries statistics of fMRI scans. The technique is akin to principal component analysis (PCA), but instead of iteratively extracting orthogonal dimensions of greatest variance, we extract uncorrelated dimensions of greatest test-retest reliability. The first component of an RCA subspace is the most reliable linear or nonlinear combination of the features, in the sense that the contrastive loss is minimized. For each subsequent component, we add a term to the loss function penalizing correlation with the other, already found, dimensions.

Note that RCA diverges conceptually from PCA in two important ways. First, we make components uncorrelated rather than orthogonal. This is due to the shift-invariance of the contrastive loss function—correlation is invariant to this shift, whereas the dot product is not. Second, we penalize correlation among scans (scores) in the training set, rather than in the weights (loadings). This ensures maximum separability between participants in the latent space, and also makes the technique general for both linear and non-linear models. While this makes components no longer perfectly uncorrelated in the test set, we find components are near-uncorrelated in the test set; weights are nearly uncorrelated as well, despite not optimizing for this directly in the training set or test set (Figure S3).

The input to the RCA algorithm is a dataset $D=\left(x_{1},\cdots ,x_{N}\right)$ of feature vectors $x_{i}\in R^{F}$ from each scan of each participant, where features here are the regional mean, standard deviation, or autocorrelation of the timeseries; the singular vectors of FC; movement parameters; or combinations thereof. RCA also requires a symmetric binary matrix *s* of size N×N , where an element $s_{i,j}=1$ if scans i and j come from the same participant, and 0 if they do not. In the language of contrastive learning, this groups pairs of feature vectors (scans) into positive (s=1) and negative (s=0) pairs depending on whether they belong to the same participant.

To fit RCA components, we fit a function $f:D\to {\mathbb{R}}^{N}$ for each component which optimizes the contrastive loss function. In the case of the standard linear version of RCA, f is a linear model, i.e., f(D∣w)=Dw for a weight vector w . For the nonlinear version, f is a small neural network, consisting of 10 hidden ReLU units.

For the first component k=1 , we initiate the computation of RCA components by fitting the model to the dataset D ,

$$f_{1}(D)=\text{argmi}{\text{n}}_{f}[L(f(D)|s)]$$

For higher components k>1 , we add a penalty term to assert decorrelation with earlier components. We note that our loss function is invariant to differences in absolute magnitudes of the embeddings, i.e., a constant offset would not change the loss function. Therefore, we enforce decorrelation, which is also invariant to these differences in mean, rather than orthogonality, which is not. We assert this decorrelation on the training set only, namely,

$$f_{k}(D)={\mathrm{argmin}}_{f}\left[L(f(D)|s)+c\sum_{\ell =1}^{k-1}\mathrm{corr}{\left(f(D),f_{\ell }(D)\right)}^{2}\right]$$

for some constant c . For the results shown here, we used c=0.1 .

In practice, the intractable arg min is replaced with a certain number of gradient descent steps to minimize the loss, chosen so that convergence is achieved for the contrastive loss. By repeating the procedure outlined above d times, we obtain d RCA dimensions.

We also tested a non-iterative variant of RCA which fit all components simultaneously. Without imposing orthogonality or decorrelation explicitly, we rely on an observation from deep learning (Achille and Soatto, 2018): different factors become disentangled in the representations learned by the model due to some implicit regularization enforcing each direction to extract different features from the data. We fit at most four components instead of ten (as we observed a rapid drop in the reliability of individual components as we increased beyond four components). Model fitting was accomplished in the linear case by making w a F×4 matrix instead of a F×1 vector, and in the nonlinear case by letting f(D) return a N×4 matrix instead of a N×1 vector. The rapid drop in reliability is likely related to ceiling effects, as components are already near ICC of 1.0.

RCA differs conceptually from canonical correlation analysis (CCA) and partial least squares (PLS) by using the same projection matrix on both sides and by supporting more than two observations. It differs conceptually from linear discriminant analysis (LDA) by allowing many classes to occupy the same linear dimension, enforcing clustering rather than linear separability. It shares similar goals to some eigenvector-based approaches for maximizing reliability (Cliff and Caruso, 1998; Landemard et al., 2021; Parra et al., 2019) but includes the critical nonlinear term in the loss function, which precludes a closed-form solution using SVD or other eigenvector-based methods.

### Visualization

To visualize the spatial weights of each RC, we plot on the surface of the brain the correlation of each latent with the feature from the given brain region. For instance, each latent dimension derived from lag-1 temporal autocorrelation is correlated with the lag-1 temporal autocorrelation across scans. We do this because the features are highly correlated across regions, and weights are not regularized, so they may not be representative of global patterns. Additionally, this allows non-linear networks to be visualized using the same methodology as linear networks.

### Structural and demographic prediction

We used unregularized linear regression to predict each univariate structural variable from the first 10 RCs, fitting on the training set and evaluating on the test set, the same training-test split used to perform RCA. Null distributions were computed by scrambling all scans, without regard to participant labels. To remove the linear effect of total brain volume, we performed linear regression of brain volume onto the variable of interest, and then performed regression on the residual as above.

Results were evaluated with the coefficient of determination $\left(R^{2}\right)$ , namely,

$$R^{2}=1-\frac{\sum_{i}{\left(y_{i}-f\left(x_{i}\right)\right)}^{2}}{\sum_{i}{\left(y_{i}-\bar{y}\right)}^{2}}.$$

Gender was predicted with logistic regression. To regress out brain volume, we performed linear regression of brain volume onto the independent variable first and then performed logistic regression on the residual. Results were evaluated using explained deviance definition of pseudo- $R^{2}$ , namely,

$$R_{\text{pseudo}}^{2}=1-\frac{\sum_{i}y_{i}\log\left(\frac{y_{i}}{f\left(x_{i}\right)}\right)+\left(1-y_{i}\right)\log\left(\frac{1-y_{i}}{1-f\left(x_{i}\right)}\right)}{\sum_{i}\left(y_{i}\log\left(\frac{y_{i}}{\bar{y}}\right)\right)+\left(1-y_{i}\right)\log\left(\frac{1-y_{i}}{1-\bar{y}}\right)}.$$

For each component, we predicted relative variance explained by computing the predictions above, but limited to only a single RC at a time; this is equivalent to the predictions of simple linear regression on the test set. We then divided this number by the $R^{2}$ obtained from the regression including all terms, providing a measure of the fraction of the variance explained by all RCs that could be explained by a single RC alone. (This differs from partial $R^{2}$ when multiple regressors predict the same variable.) To avoid misleading estimates caused by a small denominator, we only computed relative variance explained on feature-trait combinations that had significant $R^{2}$ with at least 5% variance explained.

To predict RCs from brain-wide measures of structure, we used ridge regression to predict the first 10 RCs from the surface area of each of our 360 parcels. We performed 5-fold cross validation within the training set to select the best penalty constant, and then fit the best of these again on the training set, evaluating fit on the test set.

To comply with the HCP reporting guidelines in displaying the relationship between BMI and movement in Figure 3, we set all histogram bins with fewer than three participants to white for the visualization only.

Since RCA components are not orthogonal, we estimated explained variance using regularized linear regression, fitting on the training set by predicting the features using the components, and then evaluating variance explained on the test set. To ensure a fair comparison, the same procedure was used for estimating variance with the PCA components.

## Supplementary Material

Supplement 1

## Data Availability

All data are from the Human Connectome Project young adult 1200 subject release (Van Essen et al., 2013). Code to perform RCA is available as the scikit-learn–compatible Python package scikit-rca, installable from pip, or from the URL https://github.com/maxweissenbacher/scikit-rca. Code to reproduce the figures is available at https://github.com/mwshinn/figures_for_rca_paper.
